# Correlation between long-term use of metformin and incidence of NAFLD among patients with type 2 diabetes mellitus: A real-world cohort study

**DOI:** 10.3389/fendo.2022.1027484

**Published:** 2022-11-30

**Authors:** Kuang-Hua Huang, Chiu-Hsiang Lee, Yih-Dih Cheng, Shuo-Yan Gau, Tung-Han Tsai, Ning-Jen Chung, Chien-Ying Lee

**Affiliations:** ^1^ Department of Health Services Administration, China Medical University, Taichung, Taiwan; ^2^ School of Nursing, Chung Shan Medical University, Taichung, Taiwan; ^3^ Department of Nursing, Chung Shan Medical University Hospital, Taichung, Taiwan; ^4^ School of Pharmacy, China Medical University, Taichung, Taiwan; ^5^ Department of Pharmacy, China Medical University Hospital, Taichung, Taiwan; ^6^ School of Medicine, Chung Shan Medical University, Taichung, Taiwan; ^7^ Department of Pharmacology, Chung Shan Medical University, Taichung, Taiwan; ^8^ Department of Pharmacy, Chung Shan Medical University Hospital, Taichung, Taiwan

**Keywords:** nonalcoholic fatty liver disease, metformin, type 2 diabetes mellitus, cumulative defined daily dose, NHIRD

## Abstract

**Background and aims:**

Studies have demonstrated that the short-term use of metformin benefits liver function among patients with type 2 diabetes mellitus (T2DM). However, few studies have reported on the effects of long-term metformin treatment on liver function or liver histology. This study investigated the correlation between metformin use and the incidence of nonalcoholic fatty liver disease (NAFLD) among patients with T2DM.

**Methods:**

This population-based study investigated the risk of NAFLD among patients with T2DM who received metformin treatment between 2001-2018. Metformin users and metformin nonusers were enrolled and matched to compare the risk of NAFLD.

**Results:**

After 3 years, the patients who received <300 cDDD of metformin and those with metformin use intensity of <10 and 10–25 DDD/month had odds ratios (ORs) of 1.11 (95% confidence interval [CI] = 1.06–1.16), 1.08 (95% CI = 1.02–1.13), and 1.18 (95% CI = 1.11–1.26) for NAFLD, respectively. Moreover, metformin users who scored high on the Diabetes Complications and Severity Index (DCSI) were at high risk of NAFLD. Patients with comorbid hyperlipidemia, hyperuricemia, obesity, and hepatitis C were also at high risk of NAFLD.

**Conclusion:**

Patients with T2DM who received metformin of <300 cDDD or used metformin at an intensity of <10 and 10–25 DDD/month were at a high risk of developing NAFLD. The results of this study also indicated that patients with T2DM receiving metformin and with high scores on the DCSI were at a high risk of developing NAFLD.

## Highlights

1. According to results from 3-year follow-up, metformin users with type 2 diabetes had an increased risk for NAFLD, with odds ratio of 1.11 (95% confidence interval [CI] = 1.06–1.16).
2. Metformin users who scored high on the Diabetes Complications and Severity Index (DCSI) were at high risk of NAFLD.

## Introduction

Nonalcoholic fatty liver disease (NAFLD) is a major public health concern worldwide because of its high prevalence. NAFLD is characterized by increased hepatic triglycerides in patients who do not consume alcohol excessively (1). NAFLD is typically classified into nonalcoholic fatty liver (NAFL) and nonalcoholic steatohepatitis (NASH); NASH is characterized by liver inflammation and hepatocyte damage due to the development of NAFLD (2). The accumulation of triglycerides within the cytoplasm of hepatocytes is a distinguishing characteristic of NAFLD (1).

The correlation between NAFLD and type 2 diabetes mellitus (T2DM) is indicated by insulin resistance (IR) and the progression of compensatory hyperinsulinemia leading to defective lipid metabolism and hepatic triglyceride accumulation (3). NAFLD is highly prevalent among patients with T2DM, accompanied by frequent incidences of obesity and IR (4). Hepatic fat accumulation among patients with T2DM is more likely to progress to NASH and fibrosis than among patients without T2DM (5). Patients with T2DM exhibit more than a twofold increase in the prevalence of NAFLD, regardless of the diagnostic method used (6).

Recent studies have reported that metformin can improve IR and hyperinsulinemia and may aid in the treatment of NAFLD (7). Evidence from animal and human studies has indicated that metformin may attenuate the onset and progression of NAFLD (8–11). Several studies have attributed the alleviating effects of metformin on NAFLD to the anti-inflammatory effects of metformin (12, 13),. However, metformin is not used for treating NAFLD because of a lack of evidence that metformin significantly improves liver histology (2, 14).

Few epidemiological studies have reported the effects of long-term metformin use on the risk of NAFLD among patients with T2DM. Therefore, we investigated whether long-term metformin use is associated with the risk of NAFLD by using the patient population in Taiwan’s National Health Insurance Research Database (NHIRD).

## Material and methods

### Data source

Secondary data analysis was performed in this study by using the Longitudinal Health Insurance Database (LHID; a subset of the NHIRD) from 2001 to 2018 released by the Health and Welfare Data Science Center, Ministry of Health and Welfare (HWDC, MOHW). The LHID is prepared from claims from Taiwan’s National Health Insurance (NHI) program that enrolls up to 99% of Taiwanese citizens. Hence, the database is a nationally representative health database for Taiwan. The data in the LHID, including detailed clinical data of outpatient visits, hospitalizations, diagnostic results, and prescriptions, have demonstrated high concordance between NHI claims records and patient self-reports (15). Therefore, the LHID was used to analyze the risk of NAFLD among patients with DM receiving metformin. The data in the LHID are anonymized, and the HWDC assigns scrambled random identification numbers to insured patients to protect their privacy. The requirement of informed consent was waived.

### Ethics approval

This study was conducted in compliance with the Declaration of Helsinki. Data used in the analysis were anonymized and released by the HWDC, MOHW, Taiwan. The HWDC assigns scrambled random identification numbers to insured patients to protect their privacy. The study was approved by the Central Regional Research Ethics Committee of China Medical University, Taiwan, as meeting all ethical criteria (No. CRREC-109-011).

### Study participants

**Figure 1:**
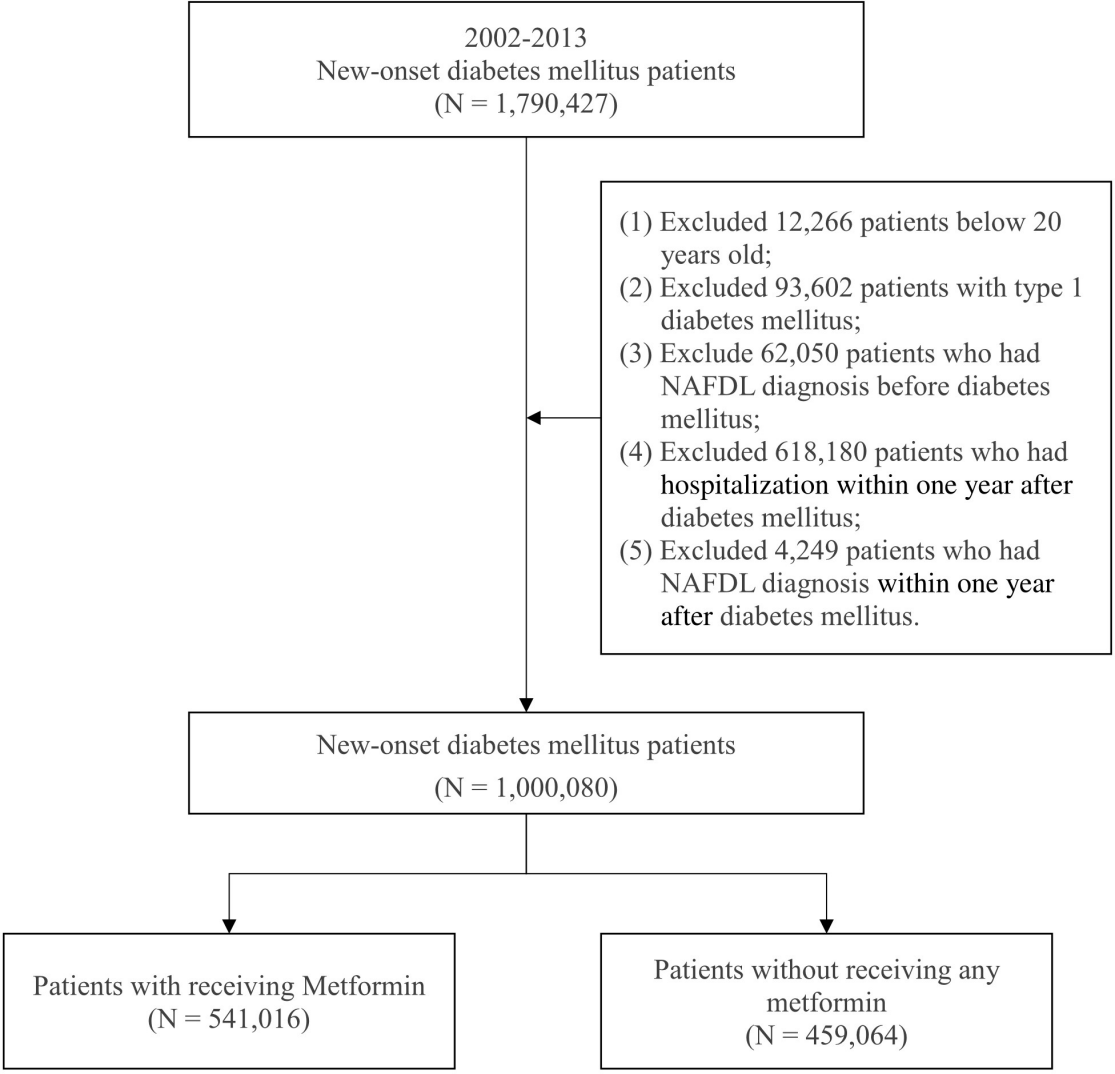
Patient selection process.

Patients with new-onset DM who were aged above 20 years were enrolled in this study to investigate the effects of metformin on incident NAFLD from 2002 to 2013. The criterion for DM was three diagnoses in a year according to the *International Classification of Diseases, Ninth Revision, Clinical Modification* (ICD-9-CM; code 250). The criterion for metformin use was based on Anatomical Therapeutic Chemical (ATC) code A10BA02. To reduce study bias, we excluded patients with type 1 DM diagnosed with NAFLD before the onset of DM, those diagnosed with NAFLD in the first year after the onset of DM, and those hospitalized within one year after the onset of DM. The patients were divided into two groups: a case group and a comparison group. The case group included patients who had received metformin in the first year after the onset of DM, and the comparison group included patients who had not received any metformin. A total of 1,000,080 patients with new-onset DM were included from 2002 to 2013; of them, 459,064 patients had not received any metformin, and 541,016 patients had received metformin in the first year after the onset of DM. **Figure 1** illustrates the process of selecting study participants.

### Study design

This study had a cross-sectional design and investigated the risk of NAFLD among patients with DM who received metformin for 3 or 5 years. The defined daily dose (DDD) is used as a standard unit for measuring drug utilization and drug exposure in a population. The World Health Organization defines DDD as the estimated average maintenance dose per day of a drug used to treat a condition in adults. The DDD does not necessarily reflect the recommended or prescribed daily dose (16). Each patient was observed for one year after the diagnosis of DM to assess the use of metformin. The DDD of metformin used to evaluate the medication was 2 g (17). The cumulative DDD (cDDD) of metformin use in the first year was calculated and categorized into five groups for dose–response analysis: nonusers, <300 cDDD, 300–500 cDDD, and above 500 cDDD. Furthermore, we calculated and categorized the average monthly DDD into four groups to investigate the association of metformin use intensity with NAFLD incidence: nonusers, <10 DDD, 10–25 DDD, and above 25 DDD. All patients were observed for 3 and 5 years to analyze the association between metformin use and NAFLD incidence. The criterion for NAFLD in this study was three or more diagnoses within one year, according to ICD-9-CM code 571.8 and ICD-10-CM codes K75.81 and K76.0. The control variables included diabetes severity and related comorbidities. We used the Diabetes Complications Severity Index (DCSI) to adjust the diabetes severity. The DCSI was used to assess the DM patients’ risks of adverse outcomes calculated by the information from the seven diabetes complication categories (retinopathy, nephropathy, neuropathy, cerebrovascular, cardiovascular, peripheral vascular disease, and metabolic) (18, 19). The assessed comorbidities included hypertension (ICD-9-CM 401–405), hyperlipidemia (ICD-9-CM 272.0–272.4), hyperuricemia (ICD-9-CM 790.6), chronic kidney disease (CKD; ICD-9-CM 585), obesity (ICD-9-CM 278.00), *Helicobacter pylori* infection (ICD-9-CM 041.86), psoriasis (ICD-9-CM 696.1), rheumatoid arthritis (RA ICD-9-CM 714), hypothyroidism (ICD-9-CM 244.9), polycystic ovary syndrome (ICD-9-CM 256.4), and hepatitis C virus (HCV; ICD-9-CM 070.4, 070.5, 070.70).

### Statistical analysis

All analyses in the study were performed using SAS version 9.4. The chi-square test was used to evaluate the distribution of the baseline characteristics between metformin users and nonusers. Differences in the incidence of NAFLD between metformin users and nonusers were estimated through multiple logistic regression with the adjustment of the relevant variables, and the results are presented as odds ratios (ORs) with 95% confidence intervals (CI). Two adjusted models were developed to estimate the incidence of NAFLD among metformin users; the estimation involved calculating the cDDD and intensity of metformin use (DDD/month). Statistical significance in this study was indicated by *p*-values <0.05.

## Results

### Characteristic distribution of study participants

**Table 1** displays the baseline characteristics of the patients. The average age of all the patients was 56.37 ± 12.49 years. Among the selected patients, 47.39% were female and 52.61% were male. Further, 29.12% of the patients were aged 20–49 years, 16.38% were aged 50–54 years, 15.77% were aged 55–59 years, 12.62% were aged 60–64 years, and 26.12% were aged ≥65 years.

**Table 1:** Baseline characteristics of patients with new-onset diabetes mellitus.

Variables	Total		Metformin				
			Non-users		Users		p-value
	N	%	N	%	N	%	
Total	1,000,080	100.00	459,064	45.90	541,016	54.10	
Gender							<0.001
Female	473,966	47.39	226,960	49.44	247,006	45.66	
Male	526,114	52.61	232,104	50.56	294,010	54.34	
Age (year) (Mean ± SD)	56.37 ± 12.49		57.92 ± 12.69		55.06 ± 12.16		<0.001
20-49	291,184	29.12	116,329	25.34	174,855	32.32	
50-54	163,773	16.38	70,456	15.35	93,317	17.25	
55-59	157,692	15.77	70,933	15.45	86,759	16.04	
60-64	126,193	12.62	60,554	13.19	65,639	12.13	
≥65	261,238	26.12	140,792	30.67	120,446	22.26	
Income level (NTD) ^a^							<0.001
≤21,000	516,216	51.62	240,725	52.44	275,491	50.92	
21,001-33,000	232,549	23.25	100,298	21.85	132,251	24.44	
≥33,001	251,315	25.13	118,041	25.71	133,274	24.63	
Urbanization ^b^							<0.001
Level 1	274,537	27.45	133,435	29.07	141,102	26.08	
Level 2	328,483	32.85	149,758	32.62	178,725	33.04	
Level 3	162,209	16.22	70,392	15.33	91,817	16.97	
Level 4	136,631	13.66	61,759	13.45	74,872	13.84	
Level 5	21,690	2.17	10,215	2.23	11,475	2.12	
Level 6	39,780	3.98	17,472	3.81	22,308	4.12	
Level 7	36,750	3.67	16,033	3.49	20,717	3.83	
DCSI score ^c^							<0.001
0	650,315	65.03	290,957	63.38	359,358	66.42	
1	195,134	19.51	90,537	19.72	104,597	19.33	
≥2	154,631	15.46	77,570	16.90	77,061	14.24	
Hypertension							<0.001
No	618,217	61.82	271,791	59.21	346,426	64.03	
Yes	381,863	38.18	187,273	40.79	194,590	35.97	
Hyperlipidemia							<0.001
No	818,863	81.88	358,393	78.07	460,470	85.11	
Yes	181,217	18.12	100,671	21.93	80,546	14.89	
Hyperuricemia							<0.001
No	992,413	99.23	454,904	99.09	537,509	99.35	
Yes	7,667	0.77	4,160	0.91	3,507	0.65	
CKD ^c^							<0.001
No	993,567	99.35	454,207	98.94	539,360	99.69	
Yes	6,513	0.65	4,857	1.06	1,656	0.31	
Obesity							<0.001
No	994,396	99.43	456,718	99.49	537,678	99.38	
Yes	5,684	0.57	2,346	0.51	3,338	0.62	
Helicobacter pylori							<0.001
No	998,245	99.82	458,068	99.78	540,177	99.84	
Yes	1,835	0.18	996	0.22	839	0.16	
Psoriasis							0.012
No	996,427	99.63	457,463	99.65	538,964	99.62	
Yes	3,653	0.37	1,601	0.35	2,052	0.38	
RA ^c^							<0.001
No	992,795	99.27	455,151	99.15	537,644	99.38	
Yes	7,285	0.73	3,913	0.85	3,372	0.62	
Hypothyroidism							<0.001
No	995,976	99.59	456,513	99.44	539,463	99.71	
Yes	4,104	0.41	2,551	0.56	1,553	0.29	
Polycystic ovary syndrome							<0.001
No	998,493	99.84	458,756	99.93	539,737	99.76	
Yes	1,587	0.16	308	0.07	1,279	0.24	
HCV ^c^							<0.001
No	996,636	99.66	457,193	99.59	539,443	99.71	
Yes	3,444	0.34	1,871	0.41	1,573	0.29	

a The premium-based salary of the patient which is according to the payroll bracket table of the National Health Insurance Administration Taiwan. NTD is New Taiwan Dollar. NTD 1 ≈ USD 0.034).

b Level 1 denoted the highest degree of urbanization, whereas level 7 denoted the lowest degree of urbanization.

c DCSI, diabetes complications severity index; CKD, chronic kidney disease; RA, rheumatoid arthritis; HCV, hepatitis C virus.

Among metformin users, the average age was 55.06 ± 12.16 years. Among the selected patients, 194,590 patients (35.97%) had hypertension, 80,546 patients (14.89%) had hyperlipidemia, 3,507 patients (0.65%) had hyperuricemia, 1,656 patients (0.31%) had CKD, 3,338 (0.62%) patients had obesity, 839 patients (0.16%) had *H. pylori* infection, 2,052 patients (0.38%) had psoriasis, 3,372 patients (0.62%) had RA, 1,553 patients (0.29%) had hypothyroidism, 1,279 patients (0.24%) had polycystic ovary syndrome, and 1,573 patients (0.29%) had HCV. Furthermore, the difference in the distribution of each comorbid disease between metformin users and nonusers was statistically significant.

### Incident NAFLD in patients with new-onset dm receiving metformin medication

**Table S1** displays the distribution of incident NAFLD among patients with T2DM. **Table 2** displays the data on NAFLD incidence obtained through 3-year follow-up; 7,451 patients (0.75%) developed NAFLD within 3 years after the diagnosis of DM. The incidence rate of NAFLD among metformin nonusers was 0.70%, and those among the metformin users were 0.79% for cDDD <300, 0.81% for cDDD 300–500, and 0.88% for cDDD ≥500. In terms of metformin use intensity, the incidence rate of NAFLD was 0.76% for <10 DDD/month, 0.85% for 10–25 DDD/month, and 0.81% for ≥25 DDD/month. After 3-year follow-up, the ORs for the incidence of NAFLD among patients with DM receiving cDDD <300, 300–500, and >500 were 1.11 (95% CI = 1.06–1.16), 1.08 (95% CI = 0.85–1.37), and 1.19 (95% CI = 0.39–3.70), respectively. In terms of metformin use intensity, the ORs for the incidence of NAFLD among patients with DM receiving <10, 10–25, and ≥25 DDD/month were 1.08 (95% CI = 1.02–1.13), 1.18 (95% CI = 1.11–1.26), and 1.09 (95% CI = 0.86–1.37), respectively. In terms of risk factors, the ORs for dementia among patients with DM scoring 1 and ≥2 on the DCSI were 1.07 (95% CI = 1.01–1.14) and 1.16 (95% CI = 1.09–1.24), respectively. Furthermore, the patients with DM comorbid with hyperlipidemia (OR = 1.30, 95% CI = 1.22–1.38) and obesity (OR = 1.78, 95% CI = 1.44–2.21) were at high risk of developing NAFLD. Patients with DM comorbid with hypertension (OR = 0.94, 95% CI = 0.89–0.99) and CKD (OR = 0.66, 95% CI = 0.47–0.94) were at low risk of developing NAFLD. By contrast, patients with comorbid hyperuricemia, *H. pylori* infection, psoriasis, RA, hypothyroidism, polycystic ovary syndrome, and HCV were not at risk of developing dementia.

**Table 2:** Three-year follow-up of incident non-alcoholic fatty liver disease in new-onset diabetes mellitus patients with metformin medication.

Variables	Three-year follow-up of incident non-alcoholic fatty liver disease												
	Events		p-value	Model 1					Model 2				
	N	%		OR	95% CI			p-value	OR	95% CI			p-value
Total	7,451	0.75											
cDDD of metformin use			<0.001										
Non-users	3,195	0.70		1					–		–		–
DDD <300	4,185	0.79		1.11	1.06	–	1.16	<0.001	–		–		–
DDD 300-500	68	0.81		1.08	0.85	–	1.37	0.535	–		–		–
DDD ≥500	3	0.88		1.19	0.39	–	3.70	0.759	–		–		–
Intensity of metformin use			<0.001										
Non-users	3,195	0.70							1				
<10	2,914	0.76		–		–		–	1.08	1.02	–	1.13	0.004
10-25	1,271	0.85		–		–		–	1.18	1.11	–	1.26	<0.001
≥25	71	0.81		–		–		–	1.09	0.86	–	1.37	0.500
Gender			<0.001										
Female	3,362	0.71		1					1				
Male	4,089	0.78		1.03	0.98	–	1.08	0.207	1.03	0.98	–	1.08	0.219
Age (year)			<0.001										
20-49	2,776	0.95		1					1				
50-54	1,232	0.75		0.79	0.73	–	0.84	<0.001	0.79	0.73	–	0.84	<0.001
55-59	1,150	0.73		0.76	0.70	–	0.81	<0.001	0.76	0.71	–	0.81	<0.001
60-64	823	0.65		0.67	0.62	–	0.73	<0.001	0.68	0.62	–	0.73	<0.001
≥65	1,470	0.56		0.58	0.55	–	0.62	<0.001	0.59	0.55	–	0.63	<0.001
Income level (NTD) ^a^			<0.001										
≤21,000	3,747	0.73		1					1				
21,001-33,000	1,646	0.71		0.94	0.89	–	1.00	0.048	0.94	0.89	–	1.00	0.049
≥33,001	2,058	0.82		1.07	1.02	–	1.14	0.012	1.07	1.02	–	1.14	0.013
Urbanization ^b^			0.429										
Level 1	2,039	0.74		1					1				
Level 2	2,518	0.77		1.04	0.98	–	1.10	0.240	1.04	0.98	–	1.10	0.237
Level 3	1,172	0.72		0.98	0.91	–	1.05	0.534	0.98	0.91	–	1.05	0.539
Level 4	1,002	0.73		1.04	0.96	–	1.12	0.361	1.04	0.96	–	1.12	0.350
Level 5	147	0.68		1.01	0.86	–	1.20	0.890	1.01	0.86	–	1.20	0.868
Level 6	285	0.72		1.05	0.93	–	1.19	0.453	1.05	0.93	–	1.19	0.438
Level 7	288	0.78		1.13	0.99	–	1.28	0.062	1.13	1.00	–	1.28	0.060
DCSI score ^c^			0.601										
0	4,805	0.74		1					1				
1	1,470	0.75		1.07	1.01	–	1.14	0.023	1.07	1.01	–	1.14	0.023
≥2	1,176	0.76		1.16	1.09	–	1.24	<0.001	1.16	1.09	–	1.24	<0.001
Hypertension			<0.001										
No	4,771	0.77		1					1				
Yes	2,680	0.70		0.94	0.89	–	0.99	0.012	0.94	0.89	–	0.99	0.011
Hyperlipidemia			<0.001										
No	5,876	0.72		1					1				
Yes	1,575	0.87		1.30	1.22	–	1.38	<0.001	1.30	1.22	–	1.38	<0.001
Hyperuricemia			0.024										
No	7,377	0.74		1					1				
Yes	74	0.97		1.23	0.97	–	1.54	0.083	1.23	0.98	–	1.55	0.081
CKD ^c^			0.017										
No	7,419	0.75		1					1				
Yes	32	0.49		0.66	0.47	–	0.94	0.022	0.66	0.47	–	0.94	0.022
Obesity			<0.001										
No	7,366	0.74		1					1				
Yes	85	1.50		1.78	1.44	–	2.21	<0.001	1.78	1.44	–	2.21	<0.001
Helicobacter pylori			0.240										
No	7,433	0.74		1					1				
Yes	18	0.98		1.19	0.75	–	1.90	0.455	1.20	0.75	–	1.91	0.452
Psoriasis			0.059										
No	7,414	0.74		1					1				
Yes	37	1.01		1.35	0.97	–	1.86	0.072	1.35	0.97	–	1.86	0.072
RA ^c^			0.756										
No	7,399	0.75		1					1				
Yes	52	0.71		1.00	0.76	–	1.32	0.979	1.01	0.77	–	1.32	0.969
Hypothyroidism			0.659										
No	7,418	0.74		1					1				
Yes	33	0.80		1.04	0.74	–	1.47	0.813	1.04	0.74	–	1.47	0.810
Polycystic ovary syndrome			0.264										
No	7,443	0.75		1					1				
Yes	8	0.50		0.50	0.25	–	1.01	0.053	0.50	0.25	–	1.01	0.054
HCV ^c^			0.946										
No	7,425	0.75		1					1				
Yes	26	0.75		1.09	0.74	–	1.61	0.649	1.10	0.75	–	1.61	0.645

a The premium-based salary of the patient which is according to the payroll bracket table of the National Health Insurance Administration Taiwan. NTD is New Taiwan Dollar. NTD 1 ≈ USD 0.034).

b Level 1 denoted the highest degree of urbanization, whereas level 7 denoted the lowest degree of urbanization.

c DCSI, diabetes complications severity index; CKD, chronic kidney disease; RA, rheumatoid arthritis; HCV, hepatitis C virus.

**Table 3** displays the 5-year follow-up data on NAFLD incidence. After adjusting the related variables, we discovered that the ORs for NAFLD incidence among patients with DM receiving cDDD <300, 300–500, and ≥500 were 1.06 (95% CI = 1.02–1.09), 0.96 (95% CI = 0.80–1.15), and 1.02 (95% CI = 0.43–2.46), respectively. In terms of the intensity of metformin use, the ORs for NAFLD incidence among patients receiving <10, 10–25, and >25 DDD/month were 1.04 (95% CI = 1.00–1.08), 1.11 (95% CI = 1.06–1.16), and 0.96 (95% CI = 0.80–1.15). Adjusted model 1 also indicated that the ORs for NAFLD incidence among patients with DM who scored 1 and ≥2 on the DCSI were 1.08 (95% CI = 1.04–1.13) and 1.14 (95% CI = 1.09–1.20), respectively. In terms of risk factors, patients with DM having hyperlipidemia (OR = 1.33, 95% CI = 1.28–1.39), hyperuricemia (OR = 1.23, 95% CI = 1.04–1.45), obesity (OR = 1.62, 95% CI = 1.38–1.91), and HCV (OR = 1.46, 95% CI = 1.15–1.86) were at high risk of developing NAFLD. Patients with comorbid hypertension (OR = 0.93, 95% CI = 0.90–0.97) and CKD (OR = 0.72, 95% CI = 0.57–0.92) were at low risk of developing NAFLD.

**Table 3:** Five-year follow-up of incident non-alcoholic fatty liver disease in new-onset diabetes mellitus patients with metformin medication.

Variables	Five-year follow-up of incident non-alcoholic fatty liver disease												
	Events		p-value	Model 1					Model 2				
	N	%		OR	95% CI			p-value	OR	95% CI			p-value
Total	14,281	1.43											
cDDD of metformin use			<0.001										
Non-users	6,294	1.37		1					–		–		–
DDD <300	7,864	1.48		1.06	1.02	–	1.09	<0.001	–		–		–
DDD 300-500	118	1.40		0.96	0.80	–	1.15	0.626	–		–		–
DDD ≥500	5	1.47		1.02	0.43	–	2.46	0.961	–		–		–
Intensity of metformin use			<0.001										
Non-users	6,294	1.37							1				
<10	5,530	1.44		–		–		–	1.04	1.00	–	1.08	0.038
10-25	2,334	1.56		–		–		–	1.11	1.06	–	1.16	<0.001
≥25	123	1.40		–		–		–	0.96	0.80	–	1.15	0.644
Gender			0.009										
Female	6,613	1.40		1					1				
Male	7,668	1.46		0.99	0.95	–	1.02	0.441	0.99	0.95	–	1.02	0.423
Age (year)			<0.001										
20-49	5,257	1.81		1					1				
50-54	2,439	1.49		0.81	0.77	–	0.85	<0.001	0.81	0.77	–	0.85	<0.001
55-59	2,213	1.40		0.76	0.72	–	0.80	<0.001	0.76	0.72	–	0.80	<0.001
60-64	1,585	1.26		0.68	0.64	–	0.72	<0.001	0.68	0.64	–	0.72	<0.001
≥65	2,787	1.07		0.58	0.55	–	0.61	<0.001	0.58	0.55	–	0.61	<0.001
Income level (NTD) ^a^			<0.001										
≤21,000	6,958	1.35		1					1				
21,001-33,000	3,344	1.44		1.03	0.98	–	1.07	0.245	1.03	0.98	–	1.07	0.239
≥33,001	3,979	1.58		1.11	1.07	–	1.16	<0.001	1.11	1.07	–	1.16	<0.001
Urbanization ^b^			0.087										
Level 1	4,013	1.46		1					1				
Level 2	4,760	1.45		1.00	0.96	–	1.04	0.961	1.00	0.96	–	1.04	0.965
Level 3	2,279	1.40		0.97	0.93	–	1.03	0.307	0.97	0.93	–	1.03	0.311
Level 4	1,906	1.39		1.01	0.96	–	1.07	0.709	1.01	0.96	–	1.07	0.695
Level 5	274	1.26		0.97	0.86	–	1.10	0.640	0.97	0.86	–	1.10	0.657
Level 6	539	1.35		1.02	0.93	–	1.12	0.624	1.02	0.94	–	1.12	0.608
Level 7	510	1.39		1.03	0.94	–	1.13	0.561	1.03	0.94	–	1.13	0.551
DCSI score ^c^			0.306										
0	9,205	1.42		1					1				
1	2,851	1.46		1.08	1.04	–	1.13	<0.001	1.08	1.04	–	1.13	0.000
≥2	2,225	1.44		1.14	1.09	–	1.20	<0.001	1.14	1.09	–	1.20	<0.001
Hypertension			<0.001										
No	9,123	1.48		1					1				
Yes	5,158	1.35		0.93	0.90	–	0.97	<0.001	0.93	0.90	–	0.97	<0.001
Hyperlipidemia			<0.001										
No	11,178	1.37		1					1				
Yes	3,103	1.71		1.33	1.28	–	1.39	<0.001	1.33	1.28	–	1.39	<0.001
Hyperuricemia			<0.001										
No	14,138	1.42		1					1				
Yes	143	1.87		1.23	1.04	–	1.45	0.015	1.23	1.04	–	1.45	0.015
CKD ^c^			0.006										
No	14,214	1.43		1					1				
Yes	67	1.03		0.72	0.57	–	0.92	0.009	0.72	0.57	–	0.92	0.009
Obesity			<0.001										
No	14,130	1.42		1					1				
Yes	151	2.66		1.62	1.38	–	1.91	<0.001	1.62	1.38	–	1.90	<0.001
Helicobacter pylori			0.083										
No	14,246	1.43		1					1				
Yes	35	1.91		1.21	0.87	–	1.69	0.262	1.21	0.87	–	1.69	0.260
Psoriasis			0.169										
No	14,219	1.43		1					1				
Yes	62	1.70		1.19	0.92	–	1.52	0.182	1.19	0.92	–	1.52	0.182
RA ^c^			0.768										
No	14,174	1.43		1					1				
Yes	107	1.47		1.07	0.88	–	1.29	0.515	1.07	0.88	–	1.29	0.507
Hypothyroidism			0.399										
No	14,216	1.43		1					1				
Yes	65	1.58		1.04	0.81	–	1.32	0.774	1.04	0.81	–	1.32	0.772
Polycystic ovary syndrome			0.777										
No	14,257	1.43		1					1				
Yes	24	1.51		0.79	0.53	–	1.19	0.262	0.80	0.53	–	1.19	0.263
HCV ^c^			0.010										
No	14,214	1.43		1					1				
Yes	67	1.95		1.46	1.15	–	1.86	0.002	1.46	1.15	–	1.86	0.002

a The premium-based salary of the patient which is according to the payroll bracket table of the National Health Insurance Administration Taiwan. NTD is New Taiwan Dollar. NTD 1 ≈ USD 0.034).

b Level 1 denoted the highest degree of urbanization, whereas level 7 denoted the lowest degree of urbanization.

c DCSI, diabetes complications severity index; CKD, chronic kidney disease; RA, rheumatoid arthritis; HCV, hepatitis C virus.

## Discussion

To the best of our knowledge, few large-scale epidemiological studies have evaluated the risk of NAFLD incidence among patients with T2DM receiving metformin. The results obtained after 3-year and 5-year follow-up indicated that patients with T2DM receiving metformin in cDDD <300 or at intensities of <10 and 10–25 DDD/month were at high risk for developing NAFLD. However, in patients with T2DM receiving metformin in cDDD of 300–500 and >500 or at intensities of >25 DDD/month, metformin exhibited no protective effects against NAFLD. In addition, metformin users who scored high on the DCSI had high ORs for NAFLD incidence among patients with T2DM; patients with comorbid hyperlipidemia, hyperuricemia, obesity and HCV were also at high risk of NAFLD.

Several studies have reported that patients with T2DM and fatty liver disease exhibited improved aminotransferase levels and IR after metformin therapy (20–23). Therefore, metformin may aid the treatment of NAFLD (8, 20, 24). Animal and physiological studies have proposed various possible mechanisms to explain the relationship between metformin use and the risk of NAFLD incidence. Metformin is considered an activator of AMP-activated protein kinase (AMPK), which is a major cellular regulator of glucose and lipid metabolism. This serves as a key mechanism through which metformin treatment aids glucose metabolism and alleviates diabetes-related complications (25). Metformin decreases triglyceride accumulation in hepatocytes due to high-fat diets *in vivo* and *in vitro* (26). Moreover, metformin can activate intracellular AMPK and stimulate NO synthesis in human aortic endothelial cells (27). The beneficial effects of metformin extend beyond glycemic control and include the improvement of hepatocyte lipid metabolism and the suppression of hepatocyte and macrophage inflammatory responses (13).

Our results indicated that in patients with T2DM receiving metformin in cDDD of 300–500 and >500 or at an intensity of >25 DDD/month, metformin exhibited no protective effects against NAFLD after 3-year and 5-year follow-up periods. Several studies have investigated the effects of metformin therapy on liver aminotransferase levels and liver histology of patients with NASH or NAFLD (10, 22, 23, 28–31). Several small open-label studies have demonstrated decreases in IR and liver aminotransferase levels with metformin use (10, 29, 31), but liver histology was not considerably improved (10, 29). Although histological necroinflammation improved among the metformin treatment group, the improvement was not statistically significant and no difference in liver fibrosis was observed between the metformin user and nonuser groups (29). Other studies have failed to demonstrate significant improvements in insulin sensitivity, aminotransferase level, or liver histology due to metformin treatment (22, 23). A meta-analysis study that included a subanalysis of the effects of metformin on biochemical and histological outcomes among NASH patients demonstrated that metformin did not improve NASH-related outcomes (28). Another meta-analysis study also demonstrated that metformin therapy did not improve liver histology among patients with NASH or NAFLD (28, 32). Therefore, the clinical administration of metformin among patients with NAFLD is limited because of mixed study results, the heterogeneous effects of treatment, and the small number of patients involved in the studies. Preclinical studies on rodents have suggested that metformin may be a useful therapeutic medication for reducing intrahepatic triacylglycerol (IHTAG) content; however, the effectiveness of metformin therapy in reducing IHTAG levels among patients has yet to be confirmed (33). Therefore, owing to a lack of evidence for significant histological improvement of the liver, metformin is not recommended for treating NASH or NAFLD in adult patients (2, 14).

Our results indicated that patients with T2DM receiving metformin in cDDD of <300 or at intensity of <10 and 10–25 DDD/month were at high risk of developing NAFLD after 3-year and 5-year follow-up periods. The effectiveness of short-term metformin treatment in reducing lipid levels and preventing lipid accumulation in hepatocytes has been frequently reported (34, 35). Metformin treatment has been reported to cause only transient improvement in liver chemistry. The reduction in insulin sensitivity due to metformin therapy was not sustainable (10). Animal and physiological studies on the effects of long-term metformin treatment have been inconclusive. Furthermore, data regarding the long-term effects of metformin therapy on liver function among patients with NAFLD are controversial. An animal study demonstrated that long-term treatment with metformin had no preventive effects against NAFLD in Zucker diabetic fatty rats (36). Studies on long-term metformin therapy have not demonstrated any histological protective effects in the liver (20–23). Moreover, metformin-induced hepatotoxic effects, including acute hepatitis, liver transaminitis, and intrahepatic cholestasis, have rarely been reported (37–40). Vitamin deficiency has been reported in many causes of chronic liver disease, and has been associated with the development of NAFLD (41). Furthermore, low vitamin B12 serum levels were revealed to be significantly correlated with NAFLD, especially in grade 2 to grade 3 hepato-steatosis (42). Another study also demonstrated that low level of vitamin B12 has been related to NAFLD patients, and the histological severity of NASH (43). Low levels of vitamin B12 have been linked to high levels of homocysteine characterizing hyper-homocysteinemia as an indicator for oxidative stress (44). Subjects with chronic liver disease can benefit from vitamin B, since its antioxidant effect has possessed hepatoprotective activity to ameliorate chronic liver injury (41). A low vitamin B12 serum level is an independent predictor of NASH histological severity and fibrosis grade (43). Serum vitamin B12 levels were significantly lower among patients with NAFLD than in controls, indicating a correlation with a higher grade of steatohepatitis (43). The prevalence of B12 deficiency was higher in metformin users than non-metformin users (45). Metformin induces vitamin B12 malabsorption may be dose-related which may increase the risk of vitamin B12 deficiency in T2DM patients (46). Several studies demonstrated that vitamin B12 deficiency occurred when patients taken metformin for more than 2-4 years (45, 47).

Metformin use is associated with vitamin B12 deficiency, which is dependent upon the cumulative dose of metformin (48). Due to the clinical benefits of metformin use, its associated side effects such as vitamin B12 deficiency is often overlooked in T2DM patients. However, the diagnosis of metformin-induced vitamin B12 deficiency may be difficult (46). Vitamin B12 deficiency play a pivotal role in the risk of NAFLD development in T2DM patients receiving cumulative dose of metformin treatment over the long term.

In summary, short-term metformin use is effective in treating NAFLD, whereas long-term cumulative dose of metformin use may not alleviate NAFLD but may instead have harmful effects. Vitamin B12 deficiency may increase the risk of NAFLD among patients with T2DM receiving cumulative dose of metformin use over the long term. However, the actual mechanism of the effects of metformin dosage on the risk of NAFLD remains unclear and should be investigated in the future. Randomized-controlled studies are warranted to verify these effects.

Our study revealed that patients with DM receiving metformin and having higher scores on the DCSI were at high risk of developing NAFLD. The prevalence of NAFLD among young adults was significantly higher than among older adults, likely because of the higher prevalence among women and metabolic syndrome among young adults (49). The DCSI is an effective tool for predicting the risk of hospitalization and mortality among patients with T2DM (18). DCSI may also be used as an indicator for estimating the risk of developing NAFLD.

The results of this study indicated that patients with DM receiving metformin with comorbid hyperlipidemia, obesity, hyperuricemia, and HCV were at high risk of developing NAFLD. Studies have demonstrated that NAFLD is a multisystem disease. Evidence indicated a strong correlation between NAFLD and increased risk of hyperlipidemia (50). Obesity is strongly correlated with the development of NAFLD (51). T2DM, IR, and obesity are key factors influencing the development of NAFLD and NASH (52). The risk of NAFLD among patients with hyperuricemia was significantly higher than among patients with normal uric acid levels (53). NAFLD is a predominant outcome of chronic HCV infection (54), which causes impairment of lipid and glucose metabolism (55).

We included data approximately covering the entire Taiwanese population in this study; thus, the sample size was large and highly representative of patients with T2DM at risk of developing NAFLD, and the data obtained were of high quality.

The follow-up period of metformin use in this study was divided into 3 years and 5 years. The cDDD of metformin use was divided into three levels: ≤300, 300–500, and >500. Similarly, the intensity of metformin use was divided into three levels, namely ≤10, 10–25, and >25 DDD/month, to investigate the correlation between T2DM and the risk of developing NAFLD.

We investigated the correlation between the risk factors of comorbidities and the risk of NAFLD incidence among patients with T2DM.

This study has several limitations that should be addressed by future studies. First, the algorithm used to categorize the severity of liver disease could not be validated because of the limitation of the NHIRD (the Child–Pugh–Turcotte score used for the prognosis of chronic liver disease was not available in the NHIRD).

Second, the ICD codes from the NHIRD data did not include detailed computed tomography findings. Third, a few factors, including alcohol consumption behavior, laboratory parameters, and abdominal ultrasonography findings, that influence NAFLD development could not be determined from the LHID, thereby affecting the findings of this study. Fourth, physical activity and eating habit are the leading causes for developing NAFDL in T2DM patients. However, we could not get information of physical activity and eating habit from these patients. Finally, although the LHID includes a large amount of data, it does not include personal information of patients, such as self-pay medical information, which could influence the development of NAFLD.

## Conclusions

Patients with T2DM who received metformin of <300 cDDD or used metformin at an intensity of <10 and 10–25 DDD/month were at a high risk of developing NAFLD. Moreover, patients receiving 300–500 and >500 cDDD of metformin or using metformin at an intensity of >25 DDD/month did not exhibit any protective effects against NAFLD.

## Data availability statement

The National Health Insurance Database used to support the findings of this study were provided by the Health and Welfare Data Science Center, Ministry of Health and Welfare (HWDC, MOHW) under license and so cannot be made freely available. Requests to access these datasets should be directed to https://dep.mohw.gov.tw/dos/np-2497-113.html.

## Ethics statement

The studies involving human participants were reviewed and approved by Central Regional Research Ethics Committee of China Medical University, Taiwan (No. CRREC-109-011). The ethics committee waived the requirement of written informed consent for participation.

## Author contributions

All the authors involved in drafting or revising the article and approved of the submitted version. Study conception and design: K-HH, C-HL, Y-DC, S-YG, T-HT, N-JC and C-YL. Data acquisition: K-HH and C-YL. Data analysis and demonstration: K-HH, T-HT and C-YL. Original draft preparation: K-HH, C-HL, Y-DC, S-YG, T-HT, N-JC and C-YL.

## Funding

This research was supported by the Chung Shan Medical University Hospital, Taiwan (CSH-2022-C-046), China Medical University Taiwan (CMU110-MF-113), and the Ministry of Science and Technology Taiwan (MOST 109-2410-H-039-004-MY2).

## Acknowledgments

Our special thanks to Chung Shan Medical University, Chung Shan Medical University Hospital, and China Medical University, which has contributed to the completion of this study. This study is based in part on data from the NHIRD. The interpretation and conclusions contained herein do not represent those of National Health Insurance Administration, Ministry of Health and Welfare or National Health Research Institutes.

## Conflict of interest

The authors declare that the research was conducted in the absence of any commercial or financial relationships that could be construed as a potential conflict of interest.

## Publisher’s note

All claims expressed in this article are solely those of the authors and do not necessarily represent those of their affiliated organizations, or those of the publisher, the editors and the reviewers. Any product that may be evaluated in this article, or claim that may be made by its manufacturer, is not guaranteed or endorsed by the publisher.
