# Supplementary material for: Correlation between long-term use of metformin and incidence of NAFLD among patients with type 2 diabetes mellitus: A real-world cohort study
Source: Front Endocrinol (Lausanne). 2022 Nov 30;13:1027484. doi: 10.3389/fendo.2022.1027484 (PMC9748475; doi:10.3389/fendo.2022.1027484)
Supplement: Supplementary file 1 [file Table_1.docx]

**Supplementary Materials**

**Table S1.** The distribution of incident non-alcoholic fatty liver disease in new-onset diabetes mellitus patients.

| Variables | Three-year follow-up | | | | Five-year follow-up | | | | |
| --- | --- | --- | --- | --- | --- | --- | --- | --- | --- |
|  | Without | | With | | Without | | With | | p-value |
|  | N | % | N | % | N | % | N | % |  |
| Total | 992,629 | 99.25 | 7,451 | 0.75 | 985,799 | 98.57 | 14,281 | 1.43 |  |
| cDDD of metformin use |  |  |  |  |  |  |  |  | <0.001 |
| Non-users | 455,869 | 99.30 | 3,195 | 0.70 | 452,770 | 98.63 | 6,294 | 1.37 |  |
| DDD <300 | 528,057 | 99.21 | 4,185 | 0.79 | 524,378 | 98.52 | 7,864 | 1.48 |  |
| DDD 300-500 | 8,366 | 99.19 | 68 | 0.81 | 8,316 | 98.60 | 118 | 1.40 |  |
| DDD >500 | 337 | 99.12 | 3 | 0.88 | 335 | 98.53 | 5 | 1.47 |  |
| Intensity of metformin use |  |  |  |  |  |  |  |  | <0.001 |
| Non-users | 455,869 | 99.30 | 3,195 | 0.70 | 452,770 | 98.63 | 6,294 | 1.37 |  |
| <10 | 379,937 | 99.24 | 2,914 | 0.76 | 377,321 | 98.56 | 5,530 | 1.44 |  |
| 10~25 | 148,120 | 99.15 | 1,271 | 0.85 | 147,057 | 98.44 | 2,334 | 1.56 |  |
| >25 | 8,703 | 99.19 | 71 | 0.81 | 8,651 | 98.60 | 123 | 1.40 |  |
| Gender |  |  |  |  |  |  |  |  | 0.009 |
| Female | 470,604 | 99.29 | 3,362 | 0.71 | 467,353 | 98.60 | 6,613 | 1.40 |  |
| Male | 522,025 | 99.22 | 4,089 | 0.78 | 518,446 | 98.54 | 7,668 | 1.46 |  |
| Age (year) |  |  |  |  |  |  |  |  | <0.001 |
| 20-49 | 288,408 | 99.05 | 2,776 | 0.95 | 285,927 | 98.19 | 5,257 | 1.81 |  |
| 50-54 | 162,541 | 99.25 | 1,232 | 0.75 | 161,334 | 98.51 | 2,439 | 1.49 |  |
| 55-59 | 156,542 | 99.27 | 1,150 | 0.73 | 155,479 | 98.60 | 2,213 | 1.40 |  |
| 66-64 | 125,370 | 99.35 | 823 | 0.65 | 124,608 | 98.74 | 1,585 | 1.26 |  |
| ≥65 | 259,768 | 99.44 | 1,470 | 0.56 | 258,451 | 98.93 | 2,787 | 1.07 |  |
| Income level (NTD) ^a^ |  |  |  |  |  |  |  |  | <0.001 |
| ≤21,000 | 512,469 | 99.27 | 3,747 | 0.73 | 509,258 | 98.65 | 6,958 | 1.35 |  |
| 21,001-33,000 | 230,903 | 99.29 | 1,646 | 0.71 | 229,205 | 98.56 | 3,344 | 1.44 |  |
| ≥33,001 | 249,257 | 99.18 | 2,058 | 0.82 | 247,336 | 98.42 | 3,979 | 1.58 |  |
| Urbanization ^b^ |  |  |  |  |  |  |  |  | 0.087 |
| Level 1 | 272,498 | 99.26 | 2,039 | 0.74 | 270,524 | 98.54 | 4,013 | 1.46 |  |
| Level 2 | 325,965 | 99.23 | 2,518 | 0.77 | 323,723 | 98.55 | 4,760 | 1.45 |  |
| Level 3 | 161,037 | 99.28 | 1,172 | 0.72 | 159,930 | 98.60 | 2,279 | 1.40 |  |
| Level 4 | 135,629 | 99.27 | 1,002 | 0.73 | 134,725 | 98.61 | 1,906 | 1.39 |  |
| Level 5 | 21,543 | 99.32 | 147 | 0.68 | 21,416 | 98.74 | 274 | 1.26 |  |
| Level 6 | 39,495 | 99.28 | 285 | 0.72 | 39,241 | 98.65 | 539 | 1.35 |  |
| Level 7 | 36,462 | 99.22 | 288 | 0.78 | 36,240 | 98.61 | 510 | 1.39 |  |
| DCSI score ^c^ |  |  |  |  |  |  |  |  | 0.306 |
| 0 | 645,510 | 99.26 | 4,805 | 0.74 | 641,110 | 98.58 | 9,205 | 1.42 |  |
| 1 | 193,664 | 99.25 | 1,470 | 0.75 | 192,283 | 98.54 | 2,851 | 1.46 |  |
| ≥2 | 153,455 | 99.24 | 1,176 | 0.76 | 152,406 | 98.56 | 2,225 | 1.44 |  |
| Hypertension |  |  |  |  |  |  |  |  | <0.001 |
| No | 613,446 | 99.23 | 4,771 | 0.77 | 609,094 | 98.52 | 9,123 | 1.48 |  |
| Yes | 379,183 | 99.30 | 2,680 | 0.70 | 376,705 | 98.65 | 5,158 | 1.35 |  |
| Hyperlipidemia |  |  |  |  |  |  |  |  | <0.001 |
| No | 812,987 | 99.28 | 5,876 | 0.72 | 807,685 | 98.63 | 11,178 | 1.37 |  |
| Yes | 179,642 | 99.13 | 1,575 | 0.87 | 178,114 | 98.29 | 3,103 | 1.71 |  |
| Hyperuricemia |  |  |  |  |  |  |  |  | <0.001 |
| No | 985,036 | 99.26 | 7,377 | 0.74 | 978,275 | 98.58 | 14,138 | 1.42 |  |
| Yes | 7,593 | 99.03 | 74 | 0.97 | 7,524 | 98.13 | 143 | 1.87 |  |
| CKD ^c^ |  |  |  |  |  |  |  |  | 0.006 |
| No | 986,148 | 99.25 | 7,419 | 0.75 | 979,353 | 98.57 | 14,214 | 1.43 |  |
| Yes | 6,481 | 99.51 | 32 | 0.49 | 6,446 | 98.97 | 67 | 1.03 |  |
| Obesity |  |  |  |  |  |  |  |  | <0.001 |
| No | 987,030 | 99.26 | 7,366 | 0.74 | 980,266 | 98.58 | 14,130 | 1.42 |  |
| Yes | 5,599 | 98.50 | 85 | 1.50 | 5,533 | 97.34 | 151 | 2.66 |  |
| Helicobacter pylori |  |  |  |  |  |  |  |  | 0.083 |
| No | 990,812 | 99.26 | 7,433 | 0.74 | 983,999 | 98.57 | 14,246 | 1.43 |  |
| Yes | 1,817 | 99.02 | 18 | 0.98 | 1,800 | 98.09 | 35 | 1.91 |  |
| Psoriasis |  |  |  |  |  |  |  |  | 0.169 |
| No | 989,013 | 99.26 | 7,414 | 0.74 | 982,208 | 98.57 | 14,219 | 1.43 |  |
| Yes | 3,616 | 98.99 | 37 | 1.01 | 3,591 | 98.30 | 62 | 1.70 |  |
| RA ^c^ |  |  |  |  |  |  |  |  | 0.768 |
| No | 985,396 | 99.25 | 7,399 | 0.75 | 978,621 | 98.57 | 14,174 | 1.43 |  |
| Yes | 7,233 | 99.29 | 52 | 0.71 | 7,178 | 98.53 | 107 | 1.47 |  |
| Hypothyroidism |  |  |  |  |  |  |  |  | 0.399 |
| No | 988,558 | 99.26 | 7,418 | 0.74 | 981,760 | 98.57 | 14,216 | 1.43 |  |
| Yes | 4,071 | 99.20 | 33 | 0.80 | 4,039 | 98.42 | 65 | 1.58 |  |
| Polycystic ovary syndrome |  |  |  |  |  |  |  |  | 0.777 |
| No | 991,050 | 99.25 | 7,443 | 0.75 | 984,236 | 98.57 | 14,257 | 1.43 |  |
| Yes | 1,579 | 99.50 | 8 | 0.50 | 1,563 | 98.49 | 24 | 1.51 |  |
| HCV ^c^ |  |  |  |  |  |  |  |  | 0.010 |
| No | 989,211 | 99.25 | 7,425 | 0.75 | 982,422 | 98.57 | 14,214 | 1.43 |  |
| Yes | 3,418 | 99.25 | 26 | 0.75 | 3,377 | 98.05 | 67 | 1.95 |  |

^a^ The premium-based salary of the patient which is according to the payroll bracket table of the National Health Insurance Administration Taiwan. NTD is New Taiwan Dollar. NTD 1 ≈ USD 0.034).

^b^ Level 1 denoted the highest degree of urbanization, whereas level 7 denoted the lowest degree of urbanization.

^c^ Abbreviations: DCSI, diabetes complications severity index; CKD, chronic kidney disease; RA, rheumatoid arthritis; HCV, hepatitis C virus.
